# Ghrelin and leptin modulate the feeding behaviour of the hawksbill turtle *Eretmochelys imbricata* during nesting season

**DOI:** 10.1093/conphys/cot016

**Published:** 2013-07-02

**Authors:** Daphne Wrobel Goldberg, Santiago Alonso Tobar Leitão, Matthew H. Godfrey, Gustave Gilles Lopez, Armando José Barsante Santos, Fabiana Alves Neves, Érica Patrícia Garcia de Souza, Anibal Sanchez Moura, Jayme da Cunha Bastos, Vera Lúcia Freire da Cunha Bastos

**Affiliations:** 1 Departamento de Bioquímica, Laboratório de Bioquímica e Toxicologia, Universidade do Estado do Rio de Janeiro, Avenida 28 de Setembro 87, Rio de Janeiro, RJ 20551-030, Brazil; 2 Fundação Pró-Tamar, Professor Ademir Francisco s/n, Barra da Lagoa, Florianópolis, SC 88061-160, Brazil; 3 NC Wildlife Resources Commission, 1507 Ann Street, Beaufort, NC 28516, USA; 4 Fundação Pró-Tamar, Caixa Postal 2219, Salvador, BA 40223-970, Brazil; 5 Fundação Pró-Tamar, Caixa Postal 50, Fernando de Noronha, PE 53990-000, Brazil; 6 Departamento de Fisiologia, Laboratório de Fisiologia da Nutrição e do Desenvolvimento, Universidade do Estado do Rio de Janeiro, Rio de Janeiro, RJ 20551-030, Brazil

**Keywords:** Fasting, migration, peptide, protein, sea turtle

## Abstract

Female sea turtles have rarely been observed foraging during the nesting season. This suggests that prior to their migration to nesting beaches the females must store sufficient energy and nutrients at their foraging grounds and must be physiologically capable of undergoing months without feeding. Leptin (an appetite-suppressing protein) and ghrelin (a hunger-stimulating peptide) affect body weight by influencing energy intake in all vertebrates. We investigated the levels of these hormones and other physiological and nutritional parameters in nesting hawksbill sea turtles in Rio Grande do Norte State, Brazil, by collecting consecutive blood samples from 41 turtles during the 2010–2011 and 2011–2012 reproductive seasons. We found that levels of serum leptin decreased over the nesting season, which potentially relaxed suppression of food intake and stimulated females to begin foraging either during or after the post-nesting migration. Concurrently, we recorded an increasing trend in ghrelin, which may have stimulated food intake towards the end of the nesting season. Both findings are consistent with the prediction that post-nesting females will begin to forage, either during or immediately after their post-nesting migration. We observed no seasonal trend for other physiological parameters (values of packed cell volume and serum levels of alanine aminotransferase, aspartate aminotransferase, alkaline phosphatase, γ-glutamyl transferase, low-density lipoprotein, and high-density lipoprotein). The observed downward trends in general serum biochemistry levels were probably due to the physiological challenge of vitellogenesis and nesting in addition to limited energy resources and probable fasting.

## Abstract (precis)

Female sea turtles rarely have been observed foraging during the nesting season. We investigated the levels of ghrelin, leptin and other physiological and nutritional parameters in nesting hawksbill sea turtles in Brazil. We found that levels of serum leptin (appetite-suppressing protein) decreased over the nesting season, while an increasing trend was observed in ghrelin (hunger-stimulating peptide). Both findings are consistent with the prediction that post-nesting females will begin to forage after the nesting season, , either during or just after their post-nesting migration.

## Introduction

Sea turtles are seasonal breeders (Hirth, 1997), and in Brazil, nesting takes place during the austral summer, between September and March (Marcovaldi *et al.*, 2007). In anticipation of the breeding season, males and females migrate hundreds or even thousands of kilometres from feeding grounds to nesting beaches (Hays *et al.*, 1999). High levels of oestrogen, secreted from the developing follicles, stimulate the production of vitellogenin, the yolk protein precursor (Heck *et al.*, 1997; Rostal *et al.*, 1998) that is synthesized by the liver, released into the bloodstream, and stored in developing oocytes (Paolucci *et al.*, 2001). Vitellogenesis in sea turtles is thought to occur 4–9 months prior to migration (Wibbels *et al.*, 1990; Kwan, 1994; Rostal *et al.*, 1998, 2001; Hamann *et al.*, 2002, 2003a), and vitellogenin levels may remain elevated for up to 5 months during this process (Heck *et al.*, 1997). However, data from the literature suggest that vitellogenesis is complete prior to the arrival of the females at the nesting grounds (Rostal *et al.*, 2001).

Hawksbill sea turtles (*Eretmochelys imbricata*) are known to nest between one and eight times within a nesting season (Chan and Liew, 1999). As the females repeatedly nest during the season, follicles are ovulated from both right and left ovaries, with a gradual decrease in the number of follicles and the size of the ovaries. Concurrently, there is a decline in circulating testosterone and estradiol (Rostal *et al.*, 1996). A reduction in testosterone and estradiol levels after each sequentially laid clutch was also observed in nesting hawksbills in Australia (Dobbs *et al.*, 2007). Female sea turtles rarely nest every year, but rather every 2 years, or more, because of the time required to build energy reserves needed for migration and reproduction (Miller, 1997; Hamann *et al.*, 2002). Additionally, they can skip reproduction when they do not have sufficient fat reserves (Broderick *et al.*, 2001). The amount of previously stored energy may allow increased investment in either the number or the quality of eggs, hence it may enhance the reproductive output of sea turtles (Hays *et al.*, 1999).

During the nesting season, most reproductive female sea turtles appear to reduce or stop eating (Bjorndal, 1985; but see Balazs, 1980; Tucker and Read, 2001). This hypophagia/aphagia has been linked to little or no availability of foraging resources in waters near the nesting beaches (Carr *et al.*, 1974), although some have noted that adequate resources exist near some nesting beaches (Delcroix *et al.*, 2009). Overall, substantial energy reserves for reproduction must be obtained prior to the turtle migrating to the nesting grounds (Hamann *et al.*, 2003b). For instance, vitellogenesis, beach emergence, oviposition, and maintenance during the internesting interval all entail high energy expenditure (Kwan, 1994). A prior study has estimated that a female hawksbill that lays three to five clutches within a season loses 11–15% of her initial body mass by the end of the nesting season (Santos *et al.*, 2010). Female sea turtles may reduce their metabolic rate and become quiescent during inter-nesting intervals in order to minimize energy expenditure (Mortimer and Portier, 1989; Hays *et al.*, 1999; McCue, 2010; Price *et al.*, 2013). According to Jones *et al.* (2009) and Price *et al.* (2013), captive green turtles decreased metabolic rate by ∼50% when fasted for 10–15 days. Sea turtle reproductive biology, combined with decreased food intake during the nesting season, can be expected to influence plasma biochemistry parameters (Honarvar *et al.*, 2011; Price *et al.*, 2013) and hormone levels, although the latter have not been well studied.

Our aim was to study hormone levels and nutritional parameters that indicate food consumption or fat metabolism in hawksbill females during the nesting season. In particular, we focused on leptin, a protein that suppresses food intake (Negrão and Licínio, 2000; Paolucci *et al.*, 2001), and ghrelin, a peptide that stimulates food uptake (Kojima *et al.*, 1999), because they affect body weight by influencing energy intake in all vertebrates (Denver *et al.*, 2011). Normally, the expression and secretion of ghrelin are increased by fasting and reduced by feeding. Conversely, leptin is an adipocyte-derived hormone related to body adiposity, and its secretion is normally reduced by fasting. In the case of reproductive female sea turtles that undergo aphagia when nesting, we expected that they would have high levels of leptin and low levels of ghrelin. An improved understanding of the interaction between food intake/energy stores and reproduction will help to inform potential conservation and management of globally endangered sea turtles (www.iucnredlist.org). For instance, this information may help managers to interpret changes in reproduction that are linked to climate change impacts on foraging grounds (Hawkes *et al.*, 2009). In addition, more complete knowledge of what constitutes a healthy sea turtle is one of the top research priorities for the conservation and management of sea turtles (Hamann *et al.*, 2010). Thus, we also describe basic physiological characteristics of reproductively active hawksbill females from Brazil.

## Materials and methods

### Study area and period

**Figure 1:**
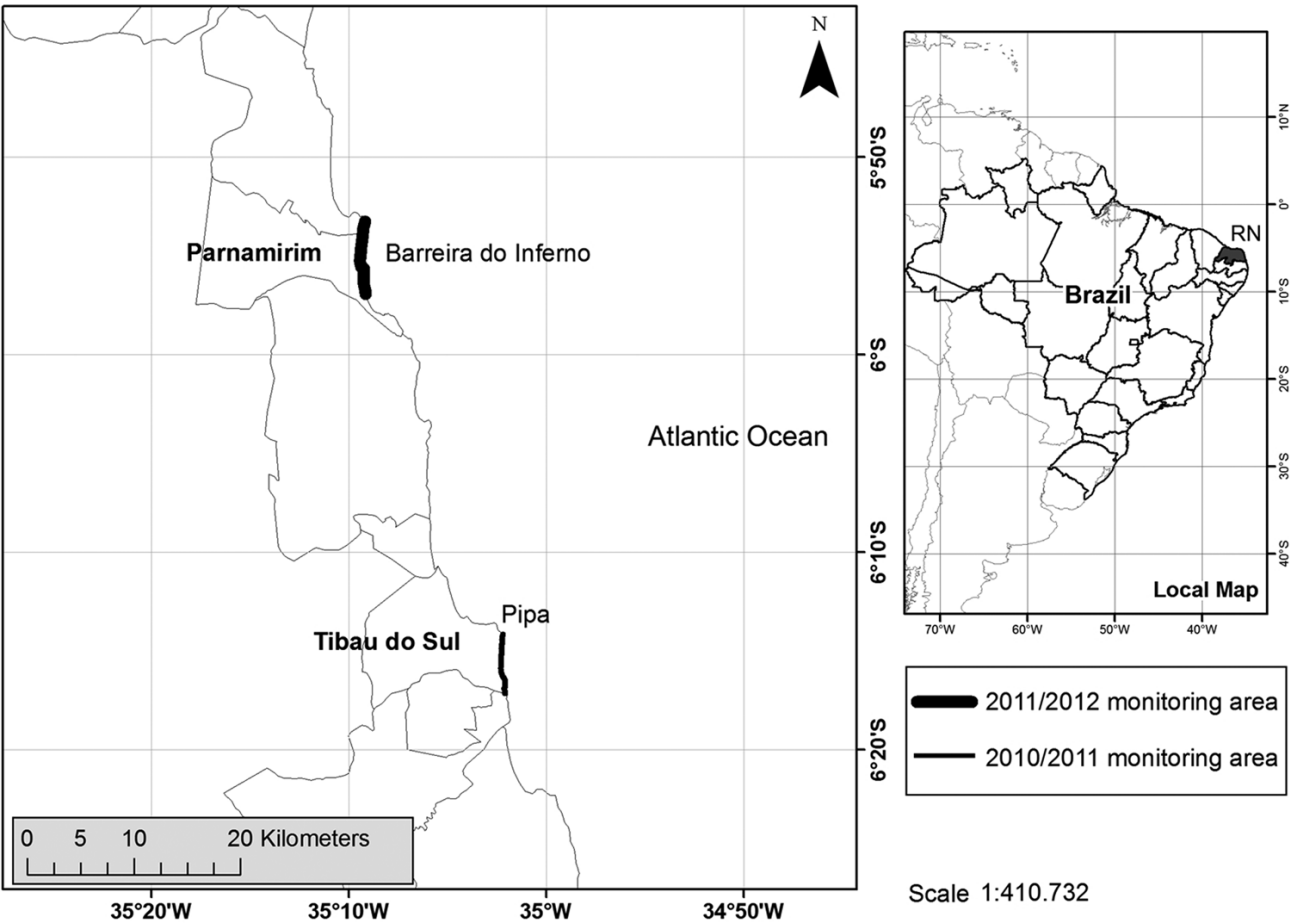
Study sites, during the 2010–2011 (thin line) and 2011–2012 nesting seasons (thick line). Both areas are located in the State of Rio Grande do Norte, Brazil.

During 2011, we collected samples from hawksbill females that were nesting on the beaches of Alagamar, Morro Branco, and Prainha in the Parnamirim municipal district, Rio Grande do Norte, Brazil (5° 54′ 56′′ S and 35° 15′ 46′′ W; Fig. 1). The area is part of the military-operated *Centro de Lançamento da Barreira do Inferno* and restricted to military activities. During 2012, we collected samples from nesting females on the beaches of Chapadão, Minas, and Sibaúma, which form part of the management area of Pipa Station (Projeto TAMAR-ICMBio). The 2012 field site is located on the southern coast of Tibau do Sul municipal district, Rio Grande do Norte State, Brazil (6° 13′ 40′′ S and 35° 03′ 05′′ W; Fig. 1).

### Blood samples

When we encountered a turtle on the nesting beach, we examined it thoroughly and applied Inconel metal tags (National Band and Tag Co., USA; style 681) on the trailing edge of the front flippers, to facilitate identification and thus to obtain consecutive blood samples from each individual. Blood samples (10 ml) were withdrawn from the dorsal cervical sinus into glass tubes with and without anticoagulant. Additionally, we recorded body mass immediately after egg laying, using a digital scale (Oswaldo Filizola, Técnica Industrial Oswaldo Filizola Ltda, São Paulo, Brazil; maximal capacity 500 kg and precision ±100 g). The accuracy of the scale was verified by weighing objects of known mass prior to and throughout data collection.

### Sample analysis

Immediately after collection, blood samples were divided into two aliquots (whole blood and heparinized plasma). Each blood aliquote was centrifuged at 1800 × *g* (4000 r.p.m.) for 10 min (model 80-2B; Centribio). The serum was separated and treated with 50 μl 1 n hydrocloric acid and 10 μl phenylmethylsulfonyl fluoride per millilitre. The packed cell volume (PCV) was determined by using a micro-haematocrit centrifuge (Model H-240, Centribio, Brasmed, Paulínia municipal district, São Paulo State, Brazil). After these procedures, samples were frozen at −20°C for subsequent analysis.

Both serum and plasma samples could be employed for all the assays carried out; however, serum was preferred because of its convenience and lower cost. Additionally, in plasma samples, the total protein is 3–5% higher due to the presence of fibrinogen (Kratz *et al.*, 2002), which may interfere with the results.

Values for very low-density lipoprotein cholesterol (VLDL) were calculated using the formula VLDL = triglyceride/5. Sodium and potassium concentrations were determined by flame photometer Corning series 400^®^. The calcium and the iron values were obtained using a Bio 2000^®^ semi-automatic analyser. The biochemical kits used for all tests were from *Bio Técnica*^®^ provided by Quality SA. Glucose concentration was measured in whole blood immediately after venipuncture, using the One Touch^®^ Ultra^®^ 2 glucometer.

As indicators of possible tissue damage, the serum activities of the following enzymes were determined: alanine aminotransferase (ALT), aspartate aminotransferase (AST), alkaline phosphatase (ALP), and γ-glutamyl transferase (GGT). These enzymes, cholesterol, urea, uric acid, and triglycerides, were processed on Ciba Express 550^®^. All samples were analysed in duplicate to monitor analytical precision.

Serum leptin and ghrelin were determined in duplicate with a commercial radioimmunoassay (RIA) kit (Linco Research, St Charles, MO, USA). The antibodies used in those kits were produced in the guinea-pig against human hormones but display cross-reactivity to leptin and ghrelin molecules of many species. Serum was used for these assays (100 μl per assay tube). We do not recommend the use of enzyme-linked immune sorbent assay kits to determine ghrelin and leptin levels. Indeed, we failed to determine the hormones levels using enzyme-linked immune sorbent assay kits because generally it requires that the antibodies recognize the N-terminal and C-terminal regions of the hormones (sandwich method). Although human and turtle ghrelin and leptin are almost identical at the N-terminal region of seven amino acids, there is much less similarity at the C-terminal region. However, the RIA kits were able to measure the levels of both hormones because the antibodies recognize only the N-terminal regions of the peptides, which are highly conserved across humans and turtles.

### Statistical analysis

The mean, SD, coefficient of variance (CV), and median were calculated for ghrelin, leptin, each serum biochemistry parameter, and the PCV value. The mean, SD, and median were also calculated for weight.

To assess whether there were trends in serum biochemistry parameters, PCV values, ghrelin and leptin levels, and weight, statistical comparisons between the first oviposition, used as a control, and subsequent nesting events within each season were carried out by means of a non-parametric Wilcoxon test. This test was conducted only on animals for which two or more nesting events were recorded. Correlations between changes in ghrelin and leptin levels and changes in weight, biochemical parameters, and PCV were tested for significance using Spearman correlations. The significance level for these tests was α = 0.05. All statistical analyses were performed using the software SPSS version 17 (SPSS Inc., Chicago, IL, USA).

## Results

Twenty nesting female hawksbill turtles were sampled in Barreira do Inferno from January to March 2011, and 21 female turtles were sampled in Pipa from January to March 2012 (total sample size = 41 individual turtles). Of all tagged turtles, 27 were sampled twice or more during separate nesting events within a season. The mean first post-oviposition mass for the 41 females was 82.2 ± 11.7 kg. For both nesting seasons, the absolute changes in mass, in relationship to the first clutch, averaged between 4.4 and 10.1 kg (Table 1).

**Table 1:** Descriptive statistics of the body mass (in kilograms) of hawksbill turtles nesting in Rio Grande do Norte State, Brazil

Descriptive statistics	Body mass according to nesting event					Variation in relationship to first nesting event			
	1st	2nd	3rd	4th	5th	2nd	3rd	4th	5th
*n*	41	27	14	7	3	27	14	7	3
Mean	82.2	78.4	80.6	80.3	81.0	−4.4	−7.8	−9.5	−10.1
SD	11.7	10.6	8.3	8.2	1.8	3.7	4.7	3.6	3.5
Minimum	60.2	58.0	65.6	63.9	79.5	−18.5	−18.3	−15.1	−13.4
First quartile	72.8	69.6	74.6	75.1	79.5	−5.6	−10.4	−11.0	−13.4
Median	82.9	79.2	84.2	83.4	80.4	−3.6	−7.1	−10.4	−10.5
Third quartile	91.8	88.4	86.2	85.6	83.0	−1.6	−4.1	−7.3	−6.4
Maximum	103.4	92.3	90.5	87.2	83.0	−0.9	−1.2	−3.8	−6.4
*P*-value for Wilcoxon test (in relationship to the first nesting event)						<0.001	<0.001	0.016	0.250^a^

Negative numbers indicate mass loss. ^a^Not significant because of the number of individuals that nested five times (*n* = 3).

The CV values for most biochemistry values were relatively low, although ghrelin and leptin had the greatest variation (Table 2).

**Table 2:** Serum biochemistry concentrations, serum hormones and packed cell volume (PCV) in hawksbill turtles (*n* = 41) nesting on Barreira do Inferno and Pipa, in Rio Grande do Norte State, Brazil

Parameter	Descriptive statistics					
	Mean	SD	CV^a^ (%)	Minimum	Median	Maximum
Triglycerides (mg/dl)	1033	202	19.6	693	989	1536
Cholesterol (mg/dl)	287	42	14.8	224	275	395
VLDL (mg/dl)	201	44	21.8	138	193	307
LDL (mg/dl)	53.8	7.1	13.2	41.0	53.0	68.0
HDL (mg/dl)	32.5	10.9	33.6	16.0	32.0	63.0
Total protein (g/dl)	5.45	0.63	11.6	4.30	5.60	6.70
Globulin (g/dl)	3.34	0.40	12.1	2.70	3.30	4.00
Albumin (g/dl)	2.11	0.43	20.5	1.30	2.10	2.80
Urea (mg/dl)	20.6	4.2	20.4	12.0	21.0	28.0
Glucose (mg/dl)	98.6	14.6	14.9	73.0	97.0	129.0
Calcium (mg/dl)	11.6	1.5	12.8	9.0	11.6	14.6
Phosphorus (mg/dl)	11.3	1.4	12.3	8.5	11.1	13.9
Sodium (mequiv/l)	139.6	3.5	2.5	132.0	140.0	145.0
Potassium (mequiv/l)	5.09	0.76	15.0	3.90	4.90	7.70
Uric acid (mg/dl)	0.95	0.17	18.4	0.60	0.90	1.30
ALT (U/l)	6.6	2.4	37.0	2.0	6.0	13.0
AST (U/l)	55.4	7.1	12.8	42.0	55.0	69.0
ALP (U/l)	15.9	3.7	23.2	7.0	16.0	24.0
GGT (U/l)	10.8	2.4	22.4	7.0	11.0	15.0
Ghrelin (pg/ml)	65.3	44.0	67.4	7.5	51.1	189.9
Leptin (ng/ml)	1070	1053	98.5	143	862	6199
PCV (%)	39.4	2.9	7.3	34.0	40.0	44.0

Biochemical concentrations are given for each turtle's first clutch of each season. Abbreviations: ALT, alanine aminotransferase; ALP, alkaline phosphatase; AST, aspartate aminotransferase; GGT, γ-glutamyl transferase; HDL, high-density lipoprotein; LDL, low-density lipoprotein; PCV, packed cell volume; and VLDL, very low-density lipoprotein. ^a^CV is the coefficient of variance = SD/mean.

**Figure 2:**
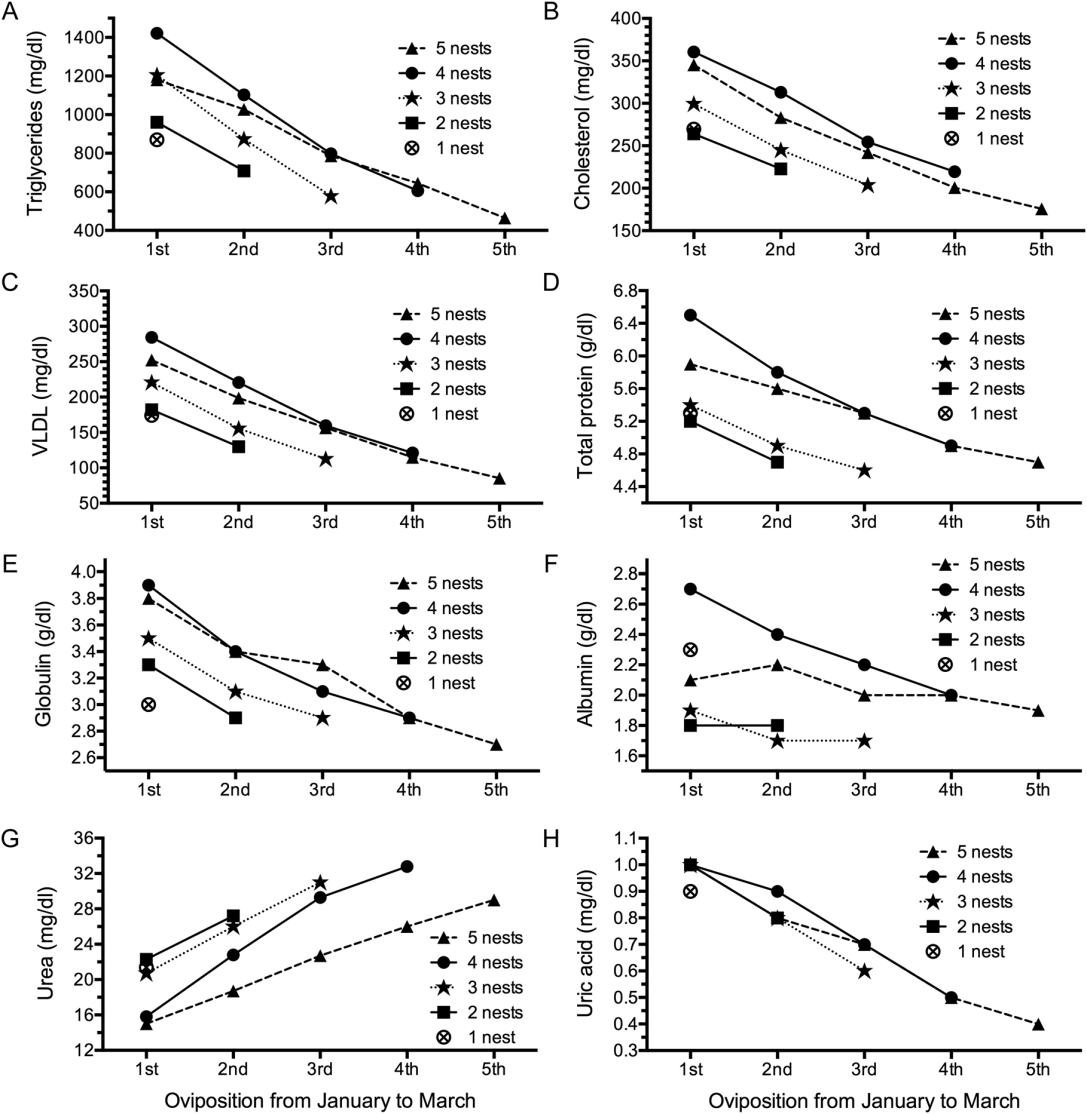
Changes in mean values of biochemical parameters, grouped by total number of nests observed during the season, in nesting hawksbill turtles in Rio Grande do Norte, Brazil. The values for triglycerides (**A**), cholesterol (**B**), very low-density lipoprotein (VLDL; **C**), total protein (**D**), globulin (**E**), albumin (**F**), urea (**G**), and uric acid (**H**) varied significantly with an increasing number of nesting events (*P* < 0.05 by Wilcoxon test for all groups, except for the turtles that nested once). Sample sizes are as follows: five nests, *n* = 3; four nests, *n* = 4; three nests, *n* = 7; two nests, *n* = 13; and one nest, *n* = 14.

A significant downward trend during subsequent nesting events was observed in the following biochemical parameters: triglycerides, cholesterol, VLDL, total protein, globulin, albumin, and uric acid. Serum urea showed an increasing trend. Statistical comparisons between the first oviposition, used as a control, and subsequent nesting events within each season were carried out by means of a non-parametric Wilcoxon test (Fig. 2A–H). The test was significant for all turtles (*P* < 0.05) except for the ones that nested five times, owing to the small sample size (*n* = 3).

**Figure 3:**
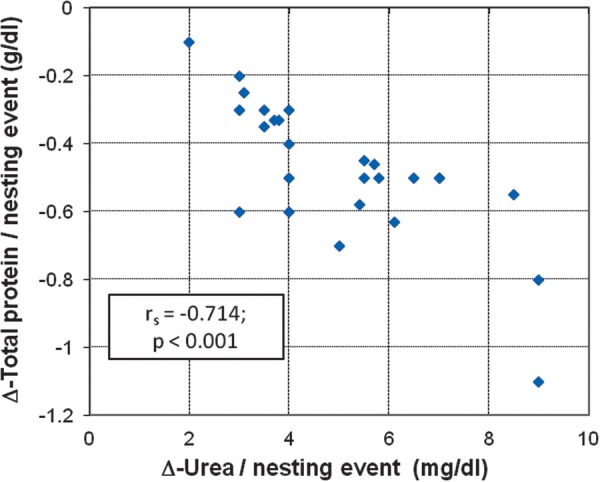
Scatter plot and Spearman rank correlation between the rates (Δ) of urea and total protein per nesting event for reproductive female hawksbill turtles observed nesting twice or more in a single season (*n* = 27). The scatter graph shows a negative correlation between the variables; urea tends to increase as total protein decreases, which indicates muscle protein catabolism throughout nesting period. Rates (Δ) were obtained from the slope of the regression line, with the number of nesting events varying from two to five times within a nesting season.

A Spearman rank correlation was conducted between the values of Δ (obtained from the slope of the regression line, with number of nesting events varying from two to five times within a nesting season) of urea and total protein per nesting event. There was a significant negative correlation between the variables, with urea serum levels tending to increase as total protein decreased (Fig. 3), which indicates muscle protein catabolism throughout the nesting period.

**Figure 4:**
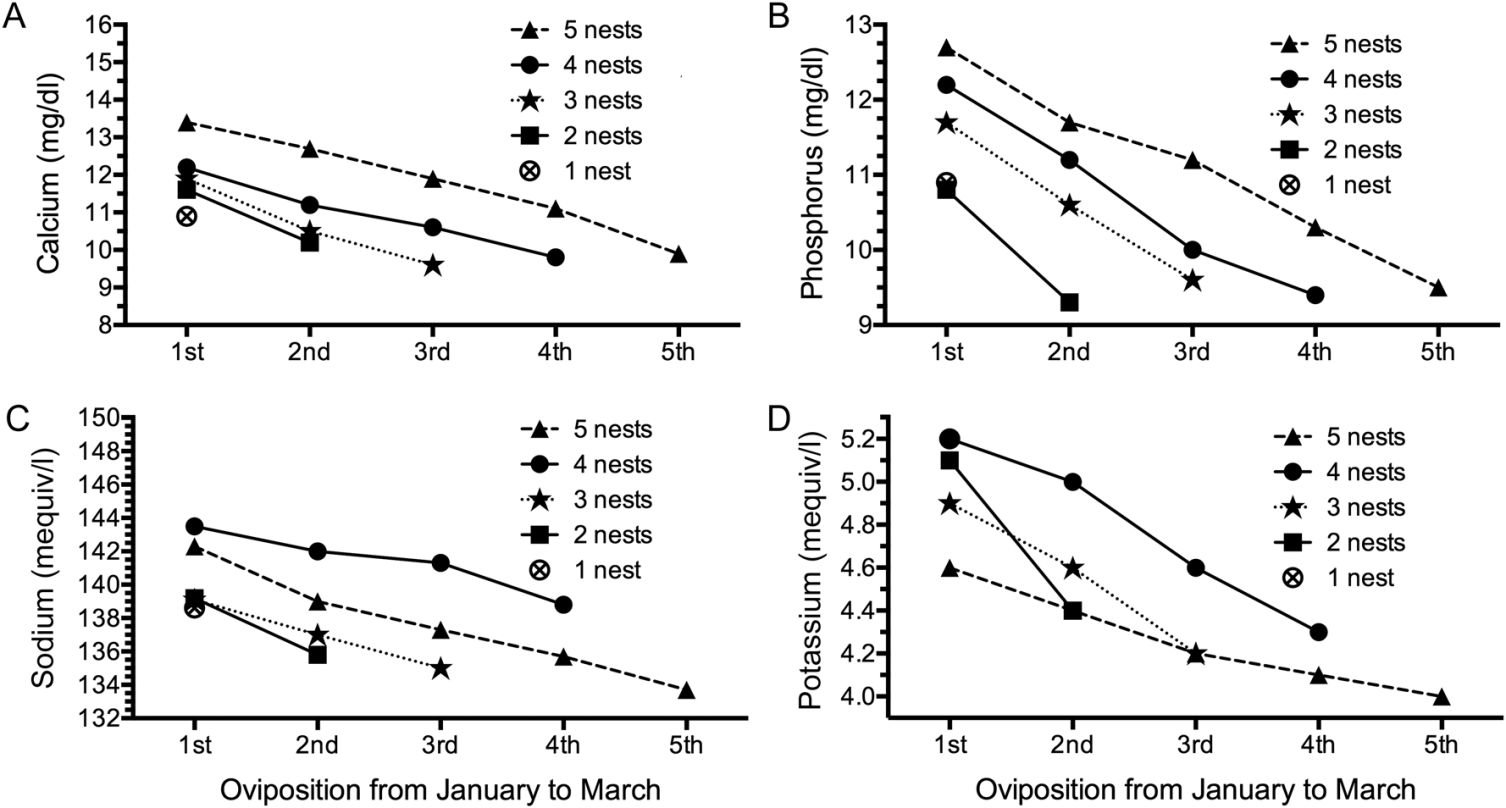
Changes in mean electrolyte values in reproductive female hawksbill turtles grouped by total number of nests observed in Rio Grande do Norte. The values for calcium (**A**), phosphorus (**B**), sodium (**C**), and potassium (**D**) decreased significantly with an increasing number of nesting events (*P* < 0.05 by Wilcoxon test for all groups, except for the turtles that nested five times and also turtles that nested once). Sample sizes are as follows: five nests, *n* = 3; four nests, *n* = 4; three nests, *n* = 7; two nests, *n* = 13; and one nest, *n* = 14.

All measured electrolytes, including calcium, phosphorus, sodium, and potassium, also decreased significantly with an increasing number of nesting events (*P* < 0.05), except for the turtles laying five clutches (*n* = 3; Fig. 4A–D).

No seasonal trend was observed for PCV values or for serum levels of ALT, AST, ALP, GGT, glucose, low-density lipoprotein (LDL), and high-density lipoprotein (HDL).

**Figure 5:**
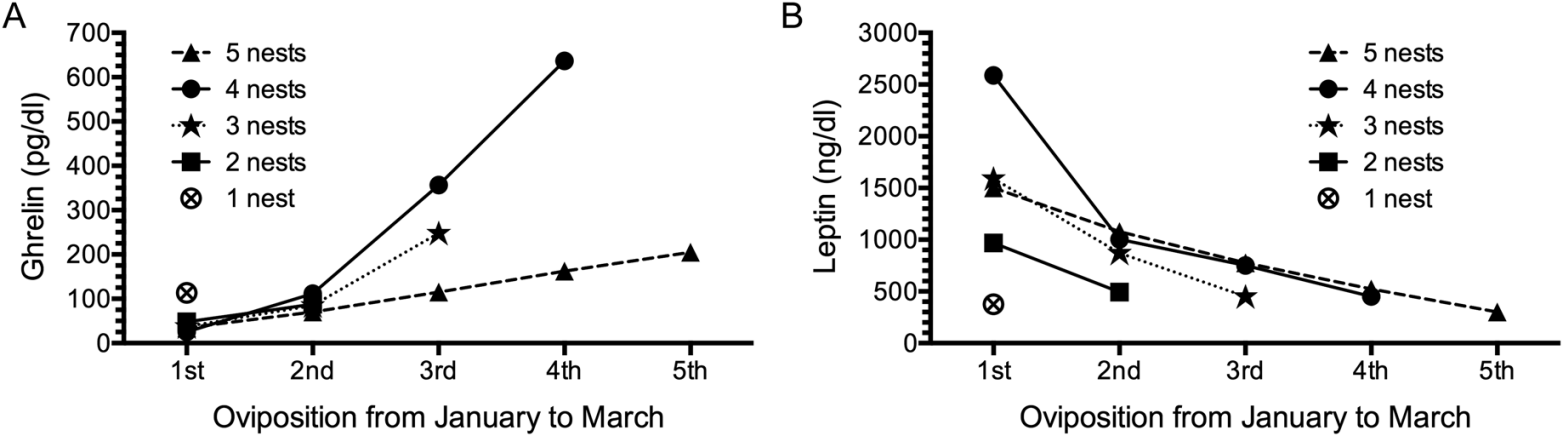
Changes in mean values of ghrelin (in picograms per millilitre) and leptin (in nanograms per millilitre) in reproductive female hawksbill turtles grouped by total number of nests observed in Rio Grande do Norte, Brazil. The values for ghrelin (**A**) increased significantly with an increasing number of nesting events, whereas the values for leptin (**B**) decreased considerably throughout the period (*P* < 0.05 by Wilcoxon test for all groups, except for the turtles that nested five times and turtles that nested once). Sample sizes are as follows: five nests, *n* = 3; four nests, *n* = 4; three nests, *n* = 7; two nests, *n* = 13; and one nest, *n* = 14.

The concentration of leptin in serum ranged between 143 and 6.199 ng/ml. The highest values were observed in the beginning of the nesting season and followed a gradual downward trend until the end of the period (Fig. 5B). The concentration of ghrelin in serum ranged between 7.5 and 189.9 pg/ml. Its serum levels were markedly lower in the beginning of the nesting season and followed an upward trend until the end of the period (Fig. 5A).

Additionally, Spearman correlations between rates of change (Δ) in serum levels of hormones (ghrelin and leptin) and in blood biochemical parameters per nesting event were conducted (Table 3).

**Table 3:** Correlations between rates of change (Δ)^a^ in serum levels of ghrelin and leptin and in blood biochemical parameters in hawksbill turtles during two or more nesting events, during 2011 and 2012 in Rio Grande do Norte, Brazil (*n* = 27)

Variation per nesting event^a^	Correlation with Δ^a^-ghrelin/nesting event		Correlation with Δ^a^-leptin/nesting event	
	Spearman coefficient (*r*_s_)	*P*-value	Spearman coefficient (*r*_s_)	*P*-value
Δ-Body mass/nesting event	−0.23	0.24	0.50	0.01*
Δ-Triglycerides/nesting event	−0.19	0.33	0.55	0.00*
Δ-Cholesterol/nesting event	−0.29	0.15	0.53	0.00*
Δ-VLDL/nesting event	−0.15	0.44	0.64	<0.001*
Δ-LDL/nesting event	−0.09	0.67	0.12	0.56
Δ-HDL/nesting event	−0.24	0.22	0.08	0.71
Δ-Total protein/nesting event	0.05	0.80	0.43	0.03*
Δ-Globulin/nesting event	0.37	0.06	0.04	0.82
Δ-Albumin/nesting event	−0.16	0.43	0.42	0.03*
Δ-Urea/nesting event	0.08	0.70	−0.29	0.14
Δ-Glucose/nesting event	−0.18	0.36	0.10	0.63
Δ-Calcium/nesting event	0.07	0.75	0.17	0.40
Δ-Phosphorus/nesting event	−0.06	0.77	0.21	0.30
Δ-Sodium/nesting event	−0.09	0.65	0.12	0.54
Δ-Potassium/nesting event	−0.26	0.19	0.11	0.57
Δ-Uric acid/nesting event	0.02	0.94	0.28	0.16
Δ-ALT/nesting event	0.04	0.85	0.07	0.71
Δ-AST/nesting event	−0.11	0.59	0.04*	0.85
Δ-ALP/nesting event	−0.35	0.07	0.30	0.12
Δ-GGT/nesting event	−0.19	0.34	0.11	0.57
Δ-PCV/nesting event	−0.14	0.48	−0.07	0.72

Abbreviations: ALT, alanine aminotransferase; ALP, alkaline phosphatase; AST, aspartate aminotransferase; GGT, γ-glutamyl transferase; HDL, high-density lipoprotein; LDL, low-density lipoprotein; PCV, packed cell volume; and VLDL, very low-density lipoprotein. ^a^Obtained from the slope of the regression line, with the number of nesting events varying from two to five times within a nesting season; negative numbers indicate a negative correlation. *Significant correlation.

## Discussion

Fasting for prolonged periods is a natural component of the life history of female sea turtles during nesting seasons. Extended fasting is characterized by mobilization and use of lipids for energy metabolism and body protein stores for gluconeogenesis. In many species, fasting occurs when the animal is engaged in other important activities that compete with feeding (Mrosovsky and Sherry, 1980). For example, sea turtle females may fast for 3 months or more during seasonal reproduction, which is also associated with high levels of energy expenditure. A recent study has estimated that nesting hawksbills from Rio Grande do Norte lose 11–15% of initial body mass by the end of the nesting season (Santos *et al.*, 2010).

### Reference intervals

To our knowledge, this is the first study in the literature to report on biochemical reference intervals for nesting hawksbills in the South Atlantic Ocean. The triglyceride concentrations (1033 ± 202 mg/dl; Table 1) were significantly higher than those found in other sea turtle species (Hasbún *et al.*, 1998; Goldberg *et al.*, 2011). Cholesterol levels (287 ± 42 mg/dl) fall within the range found by Hasbún *et al.* (1998) for female green turtles in the United Arab Emirates (226.08 ± 123.06 mg/dl) and by Goldberg *et al.* (2011) for nesting loggerheads in Brazil (247.75 ± 48.52 mg/dl). Levels of triglycerides and cholesterol are likely to be elevated in nesting females due to vitellogenesis (Derickson, 1976). Additionally, females in general may have higher mean cholesterol levels (Wilkinson, 2004).

The average values for total protein (5.45 ± 0.63 g/dl; Table 1) were slightly higher than those found by Alkindi *et al.* (2001) for nesting hawksbills in Oman (3.78 ± 0.19 g/dl) and by Deem *et al.* (2006) for nesting leatherbacks from the Republic of Gabon (4.6 ± 1.0 g/dl). Serum protein levels are often elevated during the reproductive season due to vitellogenesis, which requires increased protein synthesis (Campbell, 2004). The mean albumin values (2.11 ± 0.43 g/dl) appeared to be elevated in comparison with findings from other sea turtles (Deem *et al.*, 2006, 2009; Gelli *et al.*, 2008; Honarvar *et al.*, 2011). Female reptiles have increased albumin concentrations during vitellogenesis (Campbell, 2004), which may be associated with an increased demand for egg production. Levels of globulin were similar to those obtained by Deem *et al.* (2009) for nesting loggerheads.

Urea values ranged from 12 to 28 mg/dl (Table 1) and they were slightly lower than those reported by Campbell (2006) for adult individuals of *Caretta caretta, Chelonia mydas, Eretmochelys imbricata* and *Lepidochelys kempii* outside the reproductive period. Wyneken *et al.* (2006) reported even higher urea values for young loggerheads.

Mean uric acid values (0.95 ± 0.17 mg/dl; Table 1) were higher than the values reported by Deem *et al.* (2006) and Honarvar *et al.* (2011) for nesting leatherbacks. Moreover, uric acid levels are expected to be higher in foraging sea turtles, because the animals feed daily (Honarvar *et al.*, 2011).

The mean calcium values (11.6 ± 0.25 mg/dl; Table 1) were similar to those found by Honarvar *et al.* (2011) for reproductively active leatherbacks and slightly higher than those found by Deem *et al.* (2006) for nesting turtles of the same species. The mean phosphorus values (11.3 ± 1.4 mg/dl) fall within the range for nesting leatherbacks (Deem *et al.*, 2006; Honarvar *et al.*, 2011). Levels of both calcium and phosphorous are likely to be elevated in nesting sea turtles, owing to vitellogenesis and egg production (Wilkinson, 2004).

The mean sodium levels were 139.6 ± 3.5 mequiv/l; Table 1), similar to those in reports for nesting sea turtles (Deem *et al.*, 2006, 2009; Goldberg *et al.*, 2011; Honarvar *et al.*, 2011). Mean potassium levels were also similar to those reported in the literature for nesting sea turtles (Deem *et al.*, 2006, 2009; Honarvar *et al.*, 2011).

Relatively higher PCV values (39.4 ± 2.9%; Table 1) were found when compared with those reported by Honarvar *et al.* (2011) for nesting leatherbacks from Equatorial Guinea (36.4 ± 0.59%) and with those reported by Deem *et al.* (2006) for nesting leatherbacks (36 ± 5.4%). Fong *et al.* (2010) found even lower PCV values for adult green turtles from Taiwan. Average PCV values in juvenile hawksbills following treatment in a rehabilitation facility were also much lower (19.2 ± 3.4%; Caliendo *et al.*, 2010). Packed cell volume values have been found to increase significantly with age in loggerhead sea turtles (Kakizoe *et al.*, 2007).

The average values for ALT reported here (Table 1) fall within the range found by Honarvar *et al.* (2011) for nesting leatherbacks (8.83 ± 0.39 U/l), by Deem *et al.* (2009) for nesting loggerheads along the coast of Georgia, in the USA (4 U/l), and by Deem *et al.* (2006) for nesting leatherbacks (4 ± 2 U/l). The AST activity (55.4 ± 17.1 U/l; Table 1) was low when compared with other studies (Deem *et al.*, 2006, 2009; Goldberg *et al.*, 2011; Honarvar *et al.*, 2011). Although little is known about the tissue distribution of AST in sea turtles, AST concentrations are not considered to be organ specific in reptiles (Campbell, 2004).

Alkaline phosphatase activity (15.9 ± 3.7 U/l; Table 1) was similar to that suggested by Flint *et al.* (2010) for healthy loggerheads from Queensland, Australia and by Goldberg *et al.* (2011) for nesting loggerheads from Brazil. However, Whitaker and Krum (1999) found higher serum activities for captive juvenile loggerheads (73.9 ± 37 U/l), as did Bolten and Bjorndal (1992) for wild juvenile *C. mydas* (43 U/l ± 16). According to Campbell (2006), ALP is associated with increased osteoblastic activity. Based on this information, it is probable that animals in developmental stages show higher enzymatic levels, because osteoblasts are responsible for synthesis of bone matrix.

The serum GGT activity ranged between 7 and 15 U/l (mean 10.8 ± 2.4 U/l; Table 1). These values are similar to those reported by Deem *et al.* (2006) for nesting leatherbacks and by Deem *et al.* (2009) for nesting loggerheads, although GGT is not a parameter frequently used to evaluate health conditions in sea turtles (Wilkinson, 2004), because it is normally low (Divers, 2000).

Biochemical intervals reported here represent normal parameters for nesting hawksbills. However, there is still little published information regarding biochemical profiles of hawksbills, and further studies are urgently required.

### Serum biochemistry trends

This is the first study to evaluate trends in serum biochemistry concentrations and PCV values for nesting hawksbills. Nesting sea turtles undergo especially prolonged fasts, during which their body mass falls dramatically, and this is accompanied by predictable changes in serum biochemical concentrations.

Triglyceride and cholesterol levels as well as body mass decreased with an increasing number of nesting episodes (Fig. 2), indicating a lipolytic response to fasting. Triglycerides and cholesterol are likely to be elevated in females prior to vitellogenesis and egg formation (Derickson, 1976; Hamann *et al.*, 2003a, b; Deem *et al.*, 2009; Goldberg *et al.*, 2011). However, in the absence of food, adipose tissue triglycerides are broken down by lipase into glycerol and free fatty acids (Finn and Dice, 2006; Goldstein *et al.*, 2011; Price *et al.*, 2013). Fatty acids are used as an energy source, especially in muscle tissue, reducing the demand for organic glucose. Small amounts of glycerol from fat are then converted into glucose in the liver through gluconeogenesis (McCue, 2010). Additionally, stored triglycerides can also be used to produce ketone bodies, which are used as a glucose substitute by tissues that ordinarily require glucose (e.g. brain; Price *et al.*, 2013). Our results show that serum triglycerides were highest in turtles during the early part of the nesting season and decreased with time. This is consistent with an onset of fasting associated with the onset of nesting.

Total protein concentrations as well as albumin and globulin levels decreased significantly with the increasing number of nesting events. During fasting, catabolism of muscle proteins to amino acids produces the major source of carbon for maintenance of blood glucose levels (Finn and Dice, 2006). Liberated amino acids may provide an average calorific value of 4.4 kcal/g (Ganong, 2003). A consistent rise in blood urea throughout the nesting period (Fig. 3) also indicates utilization of muscle protein, because the first step in amino acid catabolism is removal of the amino groups, which are ultimately excreted as urea (Finn and Dice, 2006; Wood *et al.*, 2010).

Calcium, phosphorous, sodium, and potassium levels decreased significantly as the number of nesting events increased (Fig. 4). The decreasing trend in serum concentrations may represent mineral nutrient depletion as the nesting season proceeds, or it may represent an eventual return to normal (non-folliculogenic) physiological concentrations by the end of the nesting season (Honarvar *et al.*, 2011).

A decreasing trend over the course of the season was also observed in uric acid concentrations (Fig. 2). Uric acid is the primary catabolic end-product of protein and non-protein nitrogen and purines (Campbell, 2004). Food is the primary source of purines (Divers, 2000), and a decreasing trend would support extended periods of fasting during the nesting season (Honarvar *et al.*, 2011).

No seasonal trend was observed for serum glucose levels, probably because blood glucose concentration can be influenced by a wide variety of factors. For instance, during fasting, glucagon, which is stimulated by hypoglycaemia, acts in the liver to stimulate glycogenolysis and gluconeogenesis, thereby increasing blood glucose (Kaneko, 1997). Additionally, it is well established that hyperglycaemia occurs in animals during conditions of stress (Mizock, 1995), probably through enhanced catecholamine secretion, which may raise plasma glucose levels by stimulating hepatic glycogenolysis and gluconeogenesis and by interfering with peripheral tissue glucose transport (Yamada *et al.*, 1993). Hence, the stress of handling and blood collection may have induced physiological hyperglycaemia in the study animals. Aguirre *et al.* (1995) found that juvenile green turtles increased their plasma glucose concentrations in response to acute handling stress.

The downward trends in serum biochemistry levels associated with loss of body mass were probably due to the physiological stress of vitellogenesis and nesting as well as limited energy resources and possible fasting. No seasonal trend was observed for PCV values or for serum levels of ALT, AST, ALP, GGT, LDL, and HDL.

The serum levels of leptin, which inhibits food intake, decreased over the nesting season, which would potentially stimulate females to begin foraging either during or after the post-nesting migration (Fig. 5). This hormone, mainly derived from adipocytes, has been shown to circulate in concentrations proportional to body fat content (Ahren *et al.*, 1997; Paolucci *et al.*, 2001). Weight gain increases plasma leptin levels and leads to a state of negative energy balance in mammals, with energy expenditure exceeding food intake (Friedman and Halaas, 1998). If a similar cascade occurs in sea turtles, then this might be a reasonable explanation for lack of feeding during the nesting season. Conversely, food restriction decreases adiposity and lowers leptin (Negrão and Licínio, 2000), which in turn leads to a state of positive energy balance wherein food intake exceeds energy expenditure. The average values for serum leptin reported here (Table 1) were considerably higher than those found by Paolucci *et al.* (2001) for *Podarcis sicula* lizards (1.7 ng/ml) and by Krempler *et al.* (1998) for normal-weight human subjects (8.4 ng/ml). Relatively high leptin levels could be explained by the fact that sea turtles have a high body mass index and are able to store large amounts of adipose tissue under the carapace and plastron (Kwan, 1994). Unfortunately, there are no reference values for leptin levels in chelonian species. Concurrently, the increasing trend in ghrelin, which stimulates food intake, towards the end of the nesting season is consistent with the prediction that post-nesting females will begin to forage, either during or immediately after their post-nesting migration. This hormone is predominantly produced in the stomach, with fewer ghrelin-producing cells present in the large intestine and pancreas of the red-eared slider turtle, *Trachemys scripta elegans* (Kaiya *et al.*, 2004). Plasma ghrelin levels are elevated after fasting and reduced following feeding (Kojima *et al.*, 1999).

Interestingly, the mean value of serum leptin for turtles seen nesting only once was lower than for turtles seen on multiple occasions. If turtles seen only once were more likely to have been at the end of their nesting season, then we would expect them to have lower leptin than those turtles that continued to reproduce. At the same time, the mean ghrelin values for turtles seen only once were higher than for groups of turtles that were observed nesting again, consistent with our expectation above. Fasting for prolonged periods is a natural component of the life history of sea turtles, and while the associated hormonal and blood biochemistry alterations in species not adapted to extended periods of food deprivation are well defined, this is not the case for these naturally adapted reptiles.

Recently published studies indicate that leptin and ghrelin also play an important role in reproduction and may influence the levels of reproductive hormones, such as testosterone, luteinizing hormone and follicle-stimulating hormone (El-Eshmawy *et al.*, 2010). As a marker of adequate nutritional stores, these hormones may act on the central nervous system to initiate puberty and maintain normal reproductive function. In addition, leptin and ghrelin, and their receptors, are involved in reproductive events such as gonadal function and embryonic development (Budak *et al.*, 2006). However, the direct roles of ghrelin and leptin on sea turtle gonads remain unknown.

This is the first study to correlate serum levels of ghrelin and leptin with trends in serum biochemistry concentrations in nesting sea turtles. Our findings indicate that the hormones leptin and ghrelin, both capable of affecting appetite and food intake, could be involved with the hypophagia/fasting observed in nesting sea turtles. Overall, the hormonal conditions reported here are likely to be the keystone of an energetically efficient system that promotes resistance to long-term fasting in nesting grounds.

These results reinforce the hypothesis recently published by Goldstein *et al.* (2011) in which ghrelin is directly linked to the maintenance of energy metabolism during fasting conditions. Those authors showed that ghrelin is capable of raising blood glucose in mice during severe caloric restriction. Our data provide evidence that ghrelin may have a protective role in non-mammalian vertebrates (i.e. sea turtles), preventing hypoglycaemia and death during prolonged periods of fasting. The possible physiological cascade may be as follows. Fasting tends to decrease blood glucose, which causes the brain to activate the sympathetic nervous system. As a consequence, noradrenaline is released locally in the stomach wall, stimulating ghrelin secretion. Ghrelin acts in the hypothalamus and pituitary, stimulating secretion of growth hormone, which maintains blood glucose. This system becomes indispensable when adipose tissue stores have been severely depleted by chronic calorie depletion (Goldstein *et al.*, 2011). Clearly, this mechanism deserves further research; however, the study with turtles, given their robust ability to control energy metabolism, offers an excellent opportunity to understand this mechanism. Additionally, leptin and ghrelin measures may be an index of whether or not turtles are foraging at nesting sites, and could be a relatively simple yet effective way to test the hypothesis that most nesting females are not foraging.
